# Estimating the Concrete Compressive Strength Using Hard Clustering and Fuzzy Clustering Based Regression Techniques

**DOI:** 10.1155/2014/381549

**Published:** 2014-10-13

**Authors:** Naresh Kumar Nagwani, Shirish V. Deo

**Affiliations:** National Institute of Technology Raipur, Raipur, Chhattisgarh 492010, India

## Abstract

Understanding of the compressive strength of concrete is important for activities like construction arrangement, prestressing operations, and proportioning new mixtures and for the quality assurance. Regression techniques are most widely used for prediction tasks where relationship between the independent variables and dependent (prediction) variable is identified. The accuracy of the regression techniques for prediction can be improved if clustering can be used along with regression. Clustering along with regression will ensure the more accurate curve fitting between the dependent and independent variables. In this work cluster regression technique is applied for estimating the compressive strength of the concrete and a novel state of the art is proposed for predicting the concrete compressive strength. The objective of this work is to demonstrate that clustering along with regression ensures less prediction errors for estimating the concrete compressive strength. The proposed technique consists of two major stages: in the first stage, clustering is used to group the similar characteristics concrete data and then in the second stage regression techniques are applied over these clusters (groups) to predict the compressive strength from individual clusters. It is found from experiments that clustering along with regression techniques gives minimum errors for predicting compressive strength of concrete; also fuzzy clustering algorithm *C*-means performs better than *K*-means algorithm.

## 1. Introduction

Concrete is the most commonly used structural material and is composed of individual base materials [1]. Materials used in concrete, mix ratios, mixing process, transportation, and placements of concrete are all important parameters used to define concrete performance [2]. Concrete is a heterogeneous material and consists of separate phases like hydrated cement paste, transition zone, and aggregate. The compressive strength and failure of concrete are related to the weakest part of the microstructure [3]. The strength of concrete is managed by the proportioning of cement, coarse and fine aggregates, water, and various admixtures. The ratio of the water to cement is an important parameter for identifying concrete strength. Lower water-cement ratio gives higher compressive strength and minimum amount of water is necessary for the proper chemical action in the hardening of concrete, but extra water increases the workability and reduces strength of concrete [4].

A number of methods and techniques have been developed and proposed by many researchers to predict the properties of concrete. Determination of the compressive strength of concrete requires preparation, curing, and testing of special specimens [5]. The early prediction of concrete properties is an important activity, tests measuring properties of hardened concrete like strength and deformation are carried out using number of tests, and some of tests and test categorization are found in the study of Gencel et al. [6, 7]. Prediction of compressive strength of concrete is an important activity in construction technology. Timely knowledge of concrete strength helps to schedule operations such as prestressing and removal of formwork. The speed of construction can be increased using maturity methods for determining concrete compressive strength. Understanding compressive strength of concrete also helps in achieving construction quality control parameters like durability of structures and avoiding excessive loading, and so forth [8]. Regression techniques are the simplest and most efficient techniques for predicting related tasks; however, if the clustering is performed along with the regression techniques then possibly more accurate predictions can be made. Applying regression over the clustered data will identify more accurate relationships between the independent and dependent variables. In other words if regression is performed over the clustered data then more accurate curve fitting is possible between the independent and dependent variables. The objective of this work is to demonstrate that regression along with clustering gives less prediction errors for estimating compressive strength of concrete. Three regression techniques, namely, Simple Linear Regression (SLR), Logistic Regression (LR), and Least Median of Squares (LMS) along with the two popular clustering techniques *K*-means and *C*-means are used for estimating the compressive strength of concrete in this work.

## 2. Related Work

A number of works have been reported for estimation of compressive strength of the concrete and lots of recent computing techniques are used for better prediction of concrete compressive strengths. Artificial neural networks are used massively for estimating the concrete compressive strength apart from the other computing techniques. Some of the recent works related to estimating compressive strength of the concrete are discussed in this section.

### 2.1. Artificial Neural Network Based Approaches

There are numerous applications of Artificial Neural Networks (ANN) in structural engineering. ANN is used for modeling the concrete strength in a number of works [9–11]. ANNs are also used for the prediction of concrete compressive strength based on a variety of nondestructive tests [12–14]. Topçu and Saridemir [15] proposed an ANN and Fuzzy Logic based technique to determination of compressive strength of fly ash added concretes. Topçu and Saridemir (2008) [16] proposed a technique for predicting the compressive strength of concrete with some admixtures. Altun et al. (2008) [17] proposed an ANN and multiple linear regression based techniques to estimate compressive strength of steel fiber reinforced concrete. An artificial neural networks based model is used for prediction of the thermal fields in young concrete structures [1, 18]. Statistical regression analysis and artificial neural networks (ANNs) based techniques are proposed for predicting cost and schedule performance [19]. Ni and Wang [10] proposed Artificial Neural Network (ANN) and soft computing based approach for predicting concrete strength. A neural network based approach has been proposed for the evaluation of concrete compressive strength by the use of ultrasonic pulse velocity values [20]. Regression and artificial neural network (ANN) based technique is proposed for the estimation of compressive strength of vacuum processed concrete [2]. An artificial neural network of the feed-forward back-propagation type is used for the prediction of density and compressive strength properties of the cement paste portion of the concrete mixtures [3]. An artificial neural networks based technique is proposed to help concrete structure designers and engineers to compute the effects of some concrete initial parameters [21].

### 2.2. Other Computing Approaches

Fuzzy set theory has been applied in a wide range of scientific and construction research areas [15]. A number of studies are available on compressive strength, which is related to other properties or performance of concrete, like flexural strength, splitting tensile strength, elasticity modules, durability, and so forth [22–24]. A soft computing technique, namely, adaptive neurofuzzy inference system (ANFIS) is proposed by Özel [25] for predicting the compressive strength of concretes using the mix design and flow properties of concrete. Prediction of 3-day strengths of concrete compressive strength is presented by Viviani et al. [26]. A number of construction factory alternatives are proposed by Kim et al. [27] for the realization of more desirable automated construction environment. The proposed techniques are evaluated on the basis of wind speed and air temperature using computational fluid dynamics (CFD) simulation. Compressive strength computation of normal and recycled aggregate concrete is performed by Janković et al. [28] and equation for calculating compressive strength is presented. The effect of increasing the water-cement ratio over cement hydration is studied by Çolak (2006) [29]; the study can help in understanding the w/c-strength (water-cement ratio strength) relationship in concrete as the natural consequence of a progressive weakening.

An extensive study of concrete curing process is performed by Abdel-Jawad (2006) [30], where it is shown that the technical process of concrete curing involves a number of conditions for improving cement hydration. Rajamane et al. (2007) [31] analyzed the impact of temperature on strength and concluded that it depends on the time-temperature history of casting and curing; in the proposed work multivariable equations are proposed for the prediction of compressive strength of concrete. The equations given by Tanigawa et al. (1984) [32] demonstrated that prediction performance with a RMSE equal to 2.1000 can be achieved using multivariable equation technique. Classification algorithms, namely, Multilayer Perceptron, M5P Tree models and Linear Regression are used to predict the compressive strength of the high performance concrete by [4]. An estimation equation is derived for compressive strength development for the concrete containing fly ash [33]. In recent years other evolving computing techniques are also applied for predicting the compressive strength of the work.

ANNs and most of the soft computing based techniques are supervised learning techniques which require the efficient learning datasets for preparing the prediction models and always give low accuracies for prediction and other related tasks. To address this problem cluster regression based technique is proposed in this work to predict the compressive strength of concrete. The purpose of the proposed work is to demonstrate that regression technique along with clustering gives better prediction of compressive strength of the concrete. Rather than applying regression directly over the concrete dataset, it is applied over the groups (clusters) of concrete records in the proposed work. These clusters are formed on the basis of similarities between concrete records. Applying regression techniques over the clusters identifies more suitable estimations for each cluster (mathematically, better curve fitting between independent and dependent variables is achieved) which ensures the minimum forecasting errors for estimating the compressive strength of concrete.

## 3. Methodology

**Figure 1:**
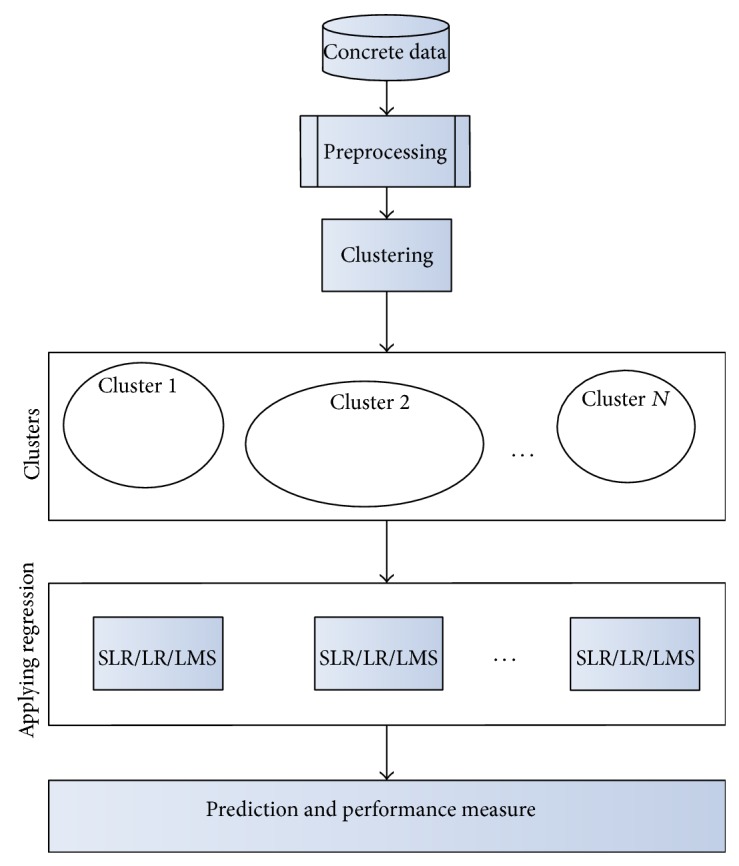
Methodology of the proposed work.

The methodology of proposed technique of estimating (predicting) the concrete compressive strength is summarized in Figure 1. The proposed method is composed of three major steps: clustering, applying regression, and performance evaluation using various parameters. These steps are summarized and explained below.

*Clustering.* In the first steps groups of similar concrete records from the concrete datasets are created. The number of groups can be decided by the user. In this study different numbers of groups are considered for the experiments. The groups are created using the clustering techniques *K*-means and fuzzy clustering technique *C*-means.

*Regression Analysis.* After creating the group of similar concrete records, regression techniques are applied to identify the relationship between concrete compressive strength with other components of concrete. In this work three regression techniques SLR, LR, and LMS are used for estimating compressive strength of concrete.

*Performance Evaluation.* Performance of prediction tasks performed in the previous steps is carried out with the help of predicting two popular error parameters MAE (Mean Absolute Error) and RMSE (Root Mean Square Error). These errors are calculated for each individual cluster and overall weighted average is also calculated for measuring the prediction errors.

Clustering is performed using *K*-means and *C*-means algorithms for creating the groups of similar characteristics concrete data. Overviews for both of these algorithms are given in this section. Distance functions play an important role in clustering, which is also discussed here in brief.

### 3.1. *K*-Means Clustering Algorithm

Clustering is a process of creating groups of similar objects. Clustering algorithms are categorized into five major categories, namely, partitioning techniques, hierarchical techniques, density based techniques, grid based techniques, and model based techniques. Partitioning techniques are the simplest techniques which create *K* number of disjoint partitions to create *K* number of clusters. These partitions are created using certain statistical measures like mean, median, and so forth. *K*-means [34] is a classical unsupervised learning algorithm used for clustering. It is a simple, low complexity, and very popular clustering algorithm.

The *K*-means algorithm [35] is a partitioning based clustering algorithm. It takes an input parameter, *K*, that is, the number of clusters to be formed, which partitions a set of *n* objects to generate the *k* clusters. The algorithm works in three steps. In the first step, *K* number of the objects are selected randomly, each of which represents the initial mean or center of the cluster. In the second step, the remaining objects are assigned to the cluster with minimum distance from cluster center or mean. In the third step, the new mean for each cluster is computed and the process iterates until the criterion function converges. The performance of *K*-means is measured using the square-error function defined in the following:

(1) $$\begin{array}{c} E=\overset{k}{\underset{i=1}{\sum }}\underset{p\in C_{i}}{\sum }{\left|p-m_{i}\right|}^{2}, \end{array}$$

where *E* is the sum of the square error, *p* is the point in space representing a given object, and *m*_*i*_ is the mean of cluster *C*_*i*_. This criterion tries to make the resulting *K* clusters as compact and as separate as possible. The algorithm is consisting of five major steps which are summarizes as given in Algorithm 1.

### 3.2. *C*-Means (Fuzzy) Clustering Algorithm

The fuzzy *C*-means (FCM) algorithm [36, 37] is one of the popularly used methods in fuzzy clustering (Algorithm 2). It is based on the concept of fuzzy *c* partitioning which is summarized as follows.

- Choose a number of clusters.
- Assign randomly to each point coefficients for being in the clusters.
- Repeat until the algorithm has converged (i.e., the coefficients' change between two iterations is no more than *ε* the given sensitivity threshold).
- Compute the centroid for each cluster, using the formula above.
- For each point, compute its coefficients of being in the clusters, using the formula above.

### 3.3. Distance Function

Clustering algorithms creates the groups of similar records. In order to find the similar records distance functions are used. Distance functions are used to measure the distance between two objects (or records). If the distance between a pair of records is less (minimum) then the records are said to be similar. In this work two clustering algorithms *K*-means and *C*-means are used to generate clusters (groups) of concrete records and Euclidian distance function is used to measure the distances between the pair of concrete records. Euclidian distance formula to measure the distance between two concrete records *C*_*i*_ and *C*_*j*_ with *N* features is presented in the following:

(2) $$\begin{array}{c} d\left(C_{i},C_{j}\right)=\sqrt{\left(\overset{N}{\underset{K=1}{\sum }}{\left|C_{i,K}-C_{j,K}\right|}^{2}\right)}. \end{array}$$

In this work UCI machine learning repository [38] of concrete data is used and there are eight (*N* = 8) attributes (features) in this dataset, namely, Cement (C), Blast Furnace Slag (BFS), Fly Ash (F), Water (W), Superplasticizer (S), Coarse Aggregate (CA), Fine Aggregate (FA), and Age (A). The description of the dataset is presented in the Table 1.

### 3.4. Regression Techniques

Regression techniques are used to discover the relationship between a set of variables. These techniques are used for identifying the patterns (relations) of independent and dependent variables, the independent variables are technically termed as response variables, and dependent variable is termed as predictors. Regression techniques typically try to relate some statistical measures like mean or average between the set of variables to identify the relationship between them. In this paper three regression techniques, namely, Simple Linear Regression (SLR), Logistic Regression (LR), and Least Median of Squares Regression technique (LMS) are used to estimate the compressive strength of concrete.

#### 3.4.1. Simple Linear Regression

Simple linear regression is the simplest regression analysis technique, where a line equation (*Y* = *c*_1_ *X*_1_ + *c*_2_ *X*_2_ + ⋯+*c*_*N*_ *X*_*N*_ + *b*) is used to relate the predictor variables (*X*_1_, *X*_2_,…, *X*_*N*_) and response to the predictor (*Y*). Simple linear regression is a statistical technique that fits a straight line to a set of (*X*, *Y*) data pairs. The slope and intercept of the fitted line are chosen so as to minimize the sum of squared differences between observed response values and fitted response values. That is, a method of ordinary least squares is used to fit a straight line model to the data.

#### 3.4.2. Logistic Regression

Logistic Regression technique is the other regression technique, which is used to perform regression on multiple independent variables presented simultaneously to predict membership of one or other of the two dependent variable categories. If the dependent variable, *Y*, is one of the binary response or dichotomous variables, logistic regression can be used to describe its relationship with several predictor variables, (*X*_1_, *X*_2_,…, *X*_*N*_), and an odds ratio can be estimated. Logistic Regression is mostly used for prediction tasks involving multiple dependent variables and is also used for exploring the strong (dominating) dependent variables in prediction tasks. Logistic Regression is used for prediction of the probability of occurrence of an event by fitting data to a logistic curve. Logistic regression techniques are designed using logistic model which can be expressed as given in the following:

(3) $$\begin{array}{c} \text{logit}\left(p\right)=\ln\frac{p}{1-p}=\alpha +b_{1}x_{1}+b_{2}x_{2}+\cdots +b_{i}x_{i}, \end{array}$$

where *p* is the probability of a classification match, and *x*_1_, *x*_2_,…, *x*_*i*_ are the explanatory, independent variables.

#### 3.4.3. Least Median of Squares (LMS) Regression

Rousseeuw (1984) [39] introduced Least Median of Squares (LMS) as a robust regression procedure. In this regression technique instead of minimizing the sum of squared residuals, coefficients are chosen so as to minimize the median of the squared residuals. In contrast to conventional least squares (LS), there is no closed-form solution with which to easily calculate the LMS line since the median is an order or rank statistic. A general nonlinear optimization algorithm performs poorly because the median of squared residuals surface is so rutted that merely local minima are often incorrectly reported as the solution.

#### 3.4.4. Regression Clustering

In regression clustering (RC) [40], regression functions are applied to the dataset simultaneously which guide the clustering of the dataset into *K* subsets each with a simpler distribution matching its guiding function. Each function is regressed on its own subset of data with a much smaller residue error. Both the regressions and the clustering optimize a common objective function.

### 3.5. Performance Measurements

The performance of regression based prediction techniques is carried in terms of errors in regression. Two such common errors in regression based prediction are Mean Absolute Error (MAE) and Root Mean Squared Error (RMSE).

#### 3.5.1. Mean Absolute Error

The Mean Absolute Error (MAE) is the average of the absolute value of the residuals (error). The MAE is very similar to the RMSE but is less sensitive to large errors. The MAE is calculated using the following:

(4) $$\begin{array}{c} \text{MAE}=\frac{1}{n}\left(\sum \left|y_{i}-{\hat{y}}_{i}\right|\right). \end{array}$$

#### 3.5.2. Root Mean Squared Error

The Root Mean Squared Error (RMSE) is the square root of the average squared distance of a data point from the fitted line. The RMSE is calculated using the following:

(5) $$\begin{array}{c} \text{RMSE}=\sqrt{\frac{\sum {\left(y_{i}-{\hat{y}}_{i}\right)}^{2}}{n}}. \end{array}$$

## 4. Concrete Compressive Strength Estimation Algorithm

Based on the methodology presented in previous section, the algorithm for predicting the compressive strength of concrete is now presented in this section (Algorithm 3). The algorithm takes two input parameters, *K*, which is the number of clusters, and *D*, a dataset containing *n* objects of Concrete samples. The algorithm is composed of five steps. In the first step the UCI machine repository concrete dataset is selected and given as input to the algorithm, and then in the second step, *K* numbers of clusters are created using *K*-means or *C*-means clustering techniques. In third step regression techniques are applied on each cluster created in step 2 to identify the relationship between the compressive strength and other concrete variables. In the fourth step, estimation of compressive strength of concrete is performed for individual clusters created in step 3 and prediction error MAE and RMSE are calculated for each cluster. Finally in the last step, weighted average (on the basis of number of concrete records belonging to various clusters) prediction errors MAE and RMSE are calculated for measuring overall errors in prediction of the compressive strength of concrete.

## 5. Experiments

### 5.1. Datasets

In order to perform the experiments of proposed technique popular dataset of compressive concrete strength from UCI machine learning repository [38] is taken. The dataset is consisting of eight input variables and one output variable, namely, “concrete compressive strength.” The dataset is summarized in Table 1, where the name of the components along with their data types and measurement units is mentioned.

### 5.2. Implementation

All the experiments are carried using Java programming language and Java based WEKA (Waikato Environment for Knowledge Analysis) tool [41]. WEKA is a popular open source tool for knowledge analysis. The implementation of *K*-means clustering technique and regression techniques is taken from the WEKA. The implementation is performed in three major steps (as shown in Figure 1). In the first step using *K*-means and *C*-means algorithms clustering is performed, where the groups of similar concrete records are created. In the second step regression techniques are applied on created clusters to identify the relationship between the dependent variable (compressive strength of concrete) with independent variables (other variables) for a cluster. In the third step, errors are calculated for the estimations. The model is thus prepared for predicting the compressive strength of concrete for the new concrete records (for which compressive strength is unknown). In case of a new concrete record, mapping of the record to a suitable cluster is carried out by measuring the belongingness to a cluster then using the equation of that cluster estimation of the compressive strength of concrete is performed.

### 5.3. Results Analysis and Discussions

**Figure 2:**
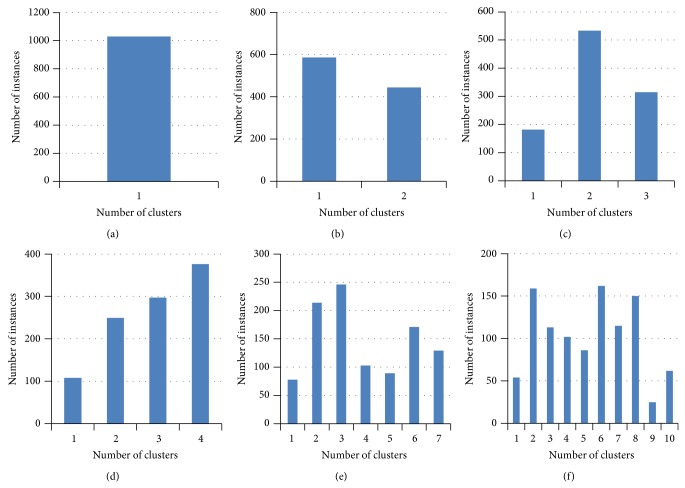
Instance distribution for different number of clusters using *K*-means algorithm.

**Figure 3:**
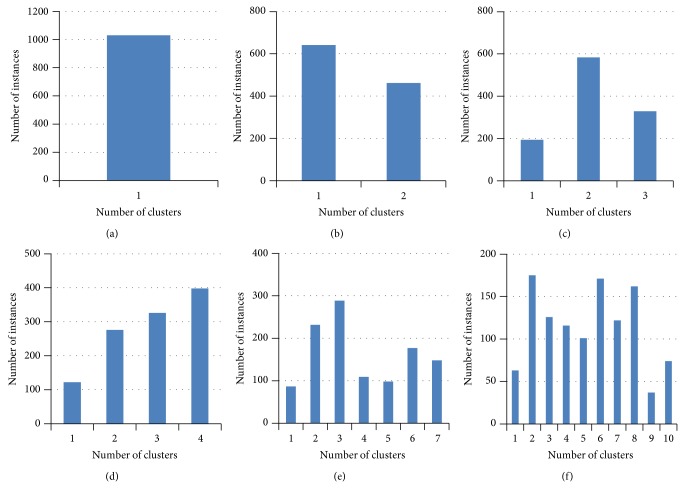
Instance distribution for different number of clusters using *C*-means algorithm.

In order to estimate the compressive strength of concrete clustering is applied (as shown in Section 3). Two clustering techniques, namely, *K*-means and *C*-means are applied to create hard and soft (fuzzy) clusters. The distribution of number of concrete instances for different number of clusters using *K*-means algorithm is shown in Figure 2. Similarly the distribution of concrete instances for *C*-means algorithm is shown in Figure 3. Since *C*-means algorithm is fuzzy technique, one instance can belong to more than one cluster hence more number of instances is belonging to the clusters than the *K*-means algorithm.

After the clustering is done, regression techniques are applied on each individual clusters for predicting the compressive strength of the concrete. Three different regression techniques SLR, LR, and LMS are applied on each individual cluster and prediction errors MAE and RMSE are recorded for each experiment. The MAE and RMSE values for each cluster using SLR regression technique is tabulated in Table 2 for *K*-means algorithm, similarly the MAE and RMSE values for clusters using LR and LMS regression techniques are tabulated in Tables 3 and 4, respectively, using the *K*-means clustering algorithm. The overall weighted MAE and overall weighted RMSE values are also recorded for each cluster. The overall weighted MAE and RMSE are calculated as follows:

(6) $$\begin{array}{c} \text{Weigthed}\;\;\text{MAE}=\frac{\sum_{i=1}^{k}\text{MA}{\text{E}}_{i}\times N_{i}}{\sum_{i=1}^{k}N_{i}},\\ \text{Weigthed}\;\;\text{RMSE}=\frac{\sum_{i=1}^{k}\text{RMS}{\text{E}}_{i}\times N_{i}}{\sum_{i=1}^{k}N_{i}}, \end{array}$$

where MAE_*i*_, RMSE_*i*_, and *N*_*i*_ are the MAE value, RMSE value, and number of concrete samples belonging to the *i*th cluster *C*_*i*_ and *k* is the total number of clusters.

The fuzzy clustering technique is based on fuzzy set theory, where the belongingness of an element in a set is decided by its degree of membership. In the fuzzy clustering technique one record may belong to several clusters at a time; therefore, for the same number of clusters more numbers of concrete records belong to the same dataset as compared with the *K*-means algorithm. The error measures MAE and RMSE for *C*-means algorithm for SLR, LR, and LMS regression techniques are tabulated in Tables 5, 6, and 7, respectively.

**Figure 4:**
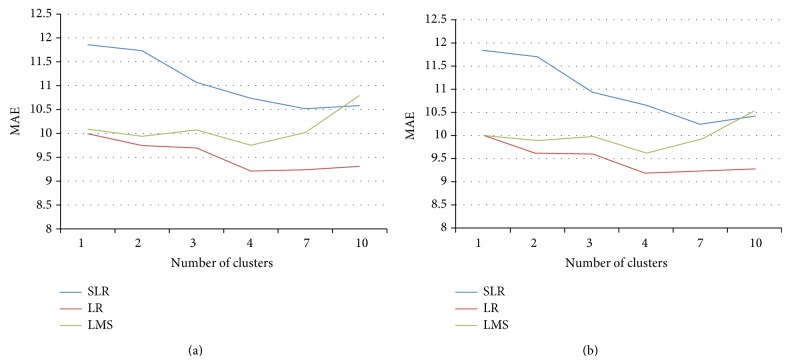
MAE values for different clusters and regression technique using (a) *K*-means and (b) *C*-means algorithms.

**Figure 5:**
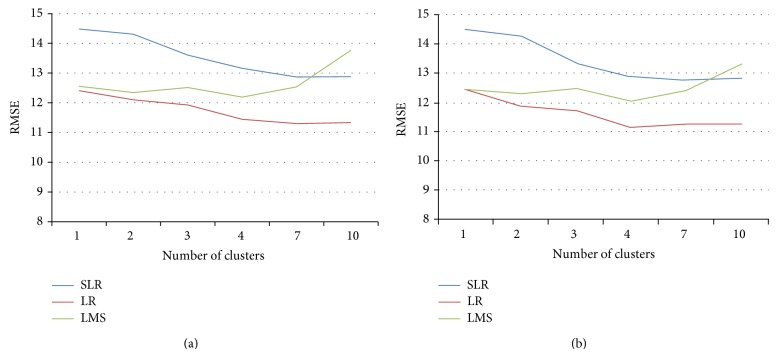
RMSE values for different clusters and regression technique using (a) *K*-means and (b) *C*-means algorithms.

The relationship between the number of concrete data clusters with overall errors MAE for *K*-means and *C*-means algorithms is shown in Figures 4(a) and 4(b), respectively. From the figures it can be seen that low values of MAE errors is found when the number of clusters are between four and seven. LR regression technique gives the minimum MAE errors in prediction. From clustering point of view minimum error is achieved by *C*-means algorithm, which is a fuzzy clustering algorithm and gives a natural way of concrete data belongingness in a particular cluster. The similar behavior is observed in Figures 5(a) and 5(b), where the relationship between RMSE values with the different number of clusters for *K*-means and *C*-means algorithm is presented. The minimum values of RMSE are achieved with the help of LR regression technique for *C*-means clustering algorithm when the numbers of clusters are between four and seven.

It is observed from the experiments that minimum errors MAE and RMSE occur when the numbers of clusters are between four and seven. LR regression technique performs better than other regression techniques and the fuzzy clustering algorithm *C*-means performs better than the *K*-means algorithm since it gives a more natural belongingness for a concrete sample to a particular cluster. So it can be concluded from the experiments that for UCI machine learning repository of concrete data the best estimation of compressive strength of concrete can be performed by clustering the concrete samples in four to seven clusters using fuzzy clustering technique followed by applying LR regression technique for estimating the concrete compressive strength for individual cluster.

From the equations generation from SLR regression technique it is found that the components C, BFS have more weights (multiplication factor) and thus are more important in estimating compressive strength of concrete, components S and FA also affects slightly in SLR regression technique. Similarly, for LR regression technique for forecasting the compressive strength of concrete the components C, BFS, W, and FA have more weights than the other components. For the LMS regression technique also the weights of components C, BFS, W, and S are more in regression equations which indicate that these components are more important than the other components for estimating the compressive strength of concrete.

## 6. Conclusion and Future Scope

In the presented work *K*-means clustering and *C*-means fuzzy clustering techniques along with the three regression techniques, namely, simple linear regression (SLR), logistic regression (LR), and least median of squares (LMS), are used to predict (estimate) the compressive strength of the concrete. The purpose of the work is to demonstrate that if clustering can be combined along with the regression technique then prediction errors for estimating compressive strength can be minimized. It is demonstrated from the experiments that if the optimum number of clusters can be created on concrete data before applying the regression then prediction errors can be minimized in efficient manners. Clustering techniques *K*-means and *C*-means are generic in nature and are capable of creating the groups objects of any data type and any number of features (attributes); hence, the same proposed model can be used for estimating compressive strength of data for different concrete datasets. In this study it is found that for UCI machine learning repository concrete data, four to seven numbers of clusters along with the regression technique give minimum prediction errors for prediction the compressive strength of concrete. It is also found that fuzzy clustering algorithm *C*-means is more efficient than the *K*-means clustering algorithm for creating the clusters and gives minimum errors in predicting the compressive strength of the concrete. Partitioning based clustering algorithms *K*-means and *C*-means are used in this work; density based clustering algorithms can be explored for creating the groups of concrete data as a future scope of the work. The proposed model can also be validated by selecting other standard datasets and comparisons can be made between partitioning based and density based clustering techniques for predicting the compressive strength of concrete.

## Figures and Tables

**Algorithm 1:**
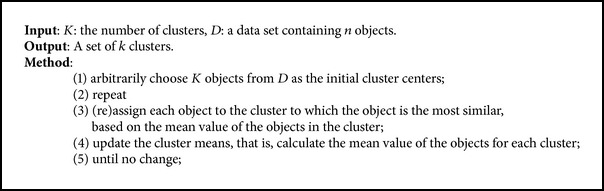
*K*-means.

**Algorithm 2:**
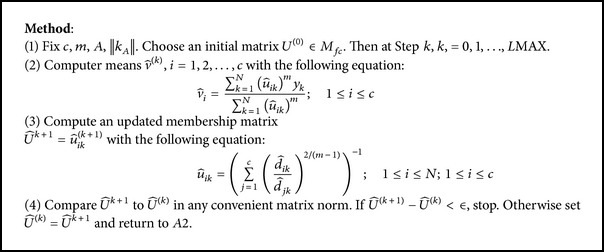
Fuzzy *C*-Means (FCM) Clustering.

**Algorithm 3:**
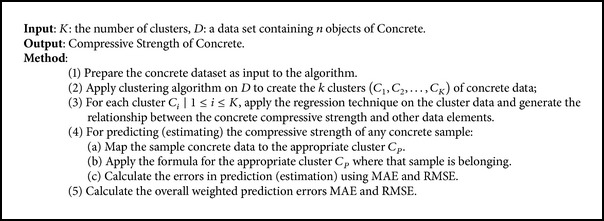
Estimation of Concrete Compressive Strength Using Cluster Regression.

**Table 1:** UCI dataset for estimating concrete compressive strength.

Name of the component	Data type	Measurement	Description	Symbol
Cement	Quantitative	kg in a m3 mixture	Input variable	**C**
Blast Furnace Slag	Quantitative	kg in a m3 mixture	Input variable	**BFS**
Fly Ash	Quantitative	kg in a m3 mixture	Input variable	**F**
Water	Quantitative	kg in a m3 mixture	Input variable	**W**
Superplasticizer	Quantitative	kg in a m3 mixture	Input variable	**S**
Coarse Aggregate	Quantitative	kg in a m3 mixture	Input variable	**CA**
Fine Aggregate	Quantitative	kg in a m3 mixture	Input variable	**FA**
Age	Quantitative	Day (1~365)	Input variable	**A**
Concrete compressive strength	Quantitative	MPa	Output variable	**CCS**

**Table 2:** MAE and RMSE using SLR regression for different number of clusters in *K*-means algorithm.

Number of clusters	Cluster number	MAE	RMSE	Number of records	Weighted MAE	Weighted RMSE
1	1	11.86	14.48	1030	11.86	14.48

2	1	12.71	15.45	586	11.73	14.31
	2	10.45	12.80	444		

3	1	12.27	14.69	182	11.07	13.60
	2	11.30	13.89	533		
	3	10.02	12.54	315		

4	1	12.01	14.37	108	10.74	13.16
	2	9.59	12.44	249		
	3	11.42	13.78	297		
	4	10.60	12.81	376		

7	1	10.86	13.31	78	**10.52**	**12.87**
	2	9.80	12.70	214		
	3	12.17	14.38	246		
	4	7.21	9.79	103		
	5	9.67	12.45	89		
	6	11.90	13.91	171		
	7	9.75	11.36	129		

10	1	12.50	14.78	54	10.58	12.88
	2	9.01	11.68	159		
	3	12.21	15.20	113		
	4	8.36	10.23	102		
	5	10.63	12.98	86		
	6	10.95	13.10	162		
	7	9.43	11.58	115		
	8	11.30	13.16	150		
	9	12.78	15.15	25		
	10	12.25	14.69	62		


**Table 3:** MAE and RMSE using Logistic regression for different number of clusters in *K*-means algorithm.

Number of clusters	Cluster number	MAE	RMSE	Number of records	Weighted MAE	Weighted RMSE
1	1	9.99	12.41	1030	9.99	12.41

2	1	10.56	13.04	586	9.75	12.10
	2	8.68	10.87	444		

3	1	11.06	13.42	182	9.69	11.93
	2	9.43	11.59	533		
	3	9.34	11.64	315		

4	1	9.41	11.23	108	**9.21**	**11.45**
	2	8.96	11.21	249		
	3	10.39	12.79	297		
	4	8.39	10.61	376		

7	1	10.70	12.49	78	**9.24**	**11.30**
	2	9.22	11.45	214		
	3	11.01	13.23	246		
	4	5.56	7.50	103		
	5	9.29	11.29	89		
	6	9.00	11.20	171		
	7	8.21	9.84	129		

10	1	10.74	13.09	54	9.31	11.33
	2	8.01	9.82	159		
	3	10.79	13.45	114		
	4	6.70	8.64	102		
	5	9.65	11.85	86		
	6	9.76	11.84	162		
	7	8.55	10.28	115		
	8	10.06	11.86	150		
	9	9.99	12.44	25		
	10	10.60	12.38	63		

**Table 4:** MAE and RMSE using LMS regression for different number of clusters in *K*-means algorithm.

Number of clusters	Cluster number	MAE	RMSE	Number of records	Weighted MAE	Weighted RMSE
1	1	10.0897	12.5563	1030	10.09	12.56

2	1	10.7509	13.23	586	9.94	12.34
	2	8.8628	11.1749	444		

3	1	11.48	14.53	182	10.07	12.52
	2	9.79	12.10	533		
	3	9.72	12.06	315		

4	1	12.17	14.58	108	**9.75**	**12.19**
	2	9.36	11.88	249		
	3	10.79	13.31	297		
	4	8.49	10.83	376		

7	1	11.23	14.50	78	10.02	12.54
	2	10.26	12.84	214		
	3	11.47	13.77	246		
	4	6.60	9.23	103		
	5	10.71	14.00	89		
	6	9.57	12.07	171		
	7	8.96	10.75	129		

10	1	12.37	15.57	54	10.80	13.77
	2	8.96	11.30	159		
	3	11.41	14.68	114		
	4	6.12	8.83	102		
	5	11.65	14.58	86		
	6	10.38	12.75	162		
	7	9.096	10.99	115		
	8	10.82	12.85	150		
	9	18.15	22.37	25		
	10	20.61	30.16	63		

**Table 5:** MAE and RMSE using SLR regression for different number of clusters in *C*-means algorithm.

Number of clusters	Cluster number	MAE	RMSE	Number of records	Weighted MAE	Weighted RMSE
1	1	11.85	14.48	1030	11.85	14.48

2	1	12.64	15.43	642	11.71	14.27
	2	10.41	12.65	462		

3	1	12.03	14.21	194	10.94	13.36
	2	11.12	13.65	583		
	3	9.98	12.34	329		

4	1	11.99	14.33	122	10.65	12.89
	2	9.56	12.24	276		
	3	11.12	13.55	326		
	4	10.62	12.35	398		

7	1	10.82	12.96	87	**10.26**	**12.78**
	2	9.77	12.76	232		
	3	11.98	14.12	289		
	4	7.11	9.76	109		
	5	9.26	12.42	98		
	6	10.97	13.88	177		
	7	9.51	11.21	148		

10	1	11.65	14.65	63	10.44	12.85
	2	8.97	11.62	175		
	3	11.95	14.98	126		
	4	8.45	10.13	116		
	5	10.22	12.92	101		
	6	10.77	12.98	171		
	7	9.56	12.24	122		
	8	11.11	12.89	162		
	9	12.65	14.78	37		
	10	11.83	14.36	74		

**Table 6:** MAE and RMSE using Logistic Regression for different number of clusters in *C*-means algorithm.

Number of clusters	Cluster number	MAE	RMSE	Number of records	Weighted MAE	Weighted RMSE
1	1	9.89	12.23	1030	9.99	12.41

2	1	10.35	12.92	642	9.60	11.9
	2	8.56	10.49	462		

3	1	10.97	13.11	194	9.61	11.71
	2	9.35	11.38	583		
	3	9.27	11.47	329		

4	1	9.25	11.01	122	**9.17**	**11.15**
	2	8.88	10.98	276		
	3	10.42	12.48	326		
	4	8.32	10.21	398		

7	1	10.61	12.44	87	**9.22**	**11.27**
	2	9.11	11.56	232		
	3	10.89	13.11	289		
	4	5.49	7.54	109		
	5	9.31	11.19	98		
	6	9.11	11.03	177		
	7	8.11	9.64	148		

10	1	10.68	12.78	63	9.26	11.21
	2	7.98	9.85	175		
	3	10.77	13.17	126		
	4	6.87	8.48	116		
	5	9.56	11.59	101		
	6	9.43	11.77	171		
	7	8.45	10.19	122		
	8	10.12	11.67	162		
	9	10.01	12.33	37		
	10	10.56	12.36	74		

**Table 7:** MAE and RMSE using LMS regression for different number of clusters in *C*-means algorithm.

Number of clusters	Cluster number	MAE	RMSE	Number of records	Weighted MAE	Weighted RMSE
1	1	10.01	12.45	1030	10.01	12.45

2	1	10.69	13.13	642	9.89	12.29
	2	8.78	11.12	462		

3	1	11.41	14.32	194	9.98	12.46
	2	9.69	12.11	583		
	3	9.65	11.98	329		

4	1	12.01	14.38	122	**9.63**	**12.06**
	2	9.22	11.68	276		
	3	10.65	13.21	326		
	4	8.34	10.67	398		

7	1	11.12	14.22	87	9.92	12.41
	2	10.11	12.48	232		
	3	11.23	13.74	289		
	4	6.49	9.14	109		
	5	10.77	13.95	98		
	6	9.54	12.03	177		
	7	8.79	10.46	148		

10	1	12.28	13.98	63	10.53	13.27
	2	8.78	11.25	175		
	3	11.26	14.59	126		
	4	5.87	8.78	116		
	5	11.43	14.23	101		
	6	10.11	11.48	171		
	7	8.84	10.23	122		
	8	10.64	14.43	162		
	9	16.49	19.78	37		
	10	18.62	24.29	74		

**Table 8:** Equations for different clusters for estimating concrete compressive strength using SLR regression.

Number of clusters	Cluster	Equation
1	1	0.08 ∗ C + 13.44

2	1	1.31 ∗ S + 32.51
	2	0.09 ∗ C + 11.77

3	1	0.07 ∗ BFS + 45.52
	2	0.06 ∗ C + 14.68
	3	0.1 ∗ C + 11.07

4	1	0.07 ∗ BFS + 46.95
	2	0.13 ∗ C − 11.73
	3	0.14 ∗ C + 2.89
	4	0.09 ∗ C + 12.45

7	1	−0.05 ∗ FA + 101.14
	2	0.12 ∗ C − 9.98
	3	0.15 ∗ C + 1.02
	4	0.1 ∗ C + 15.09
	5	0.08 ∗ C + 12.65
	6	−0.29 ∗ W + 80.13
	7	−0.41 ∗ W + 100.14

10	1	0.07 ∗ C + 24.78
	2	0.07 ∗ C + 6.25
	3	0.14 ∗ C + 3.17
	4	0.11 ∗ C + 10.4
	5	0.04 ∗ C + 27.15
	6	1.97 ∗ S + 17.71
	7	1.09 ∗ S + 25.07
	8	0.07 ∗ C + 11.93
	9	−0.64 ∗ W + 156.24
	10	0.07 ∗ C + 17.9

**Table 9:** Equations for different clusters for estimating concrete compressive strength using LR regression.

Number of clusters	Cluster	Equation
1	1	0.1067 ∗ C + 0.077 ∗ BFS + 0.0543 ∗ F + −0.1201 ∗ W + 0.301 ∗ S + 17.1208

2	1	0.1095 ∗ C + 0.0771 ∗ BFS + 0.5861 ∗ S − 7.3254
	2	0.1741 ∗ C + 0.1657 ∗ BFS + 0.1278 ∗ F + 0.2454 ∗ S + 0.0714 ∗ CA + 0.0667 ∗ FA + −153.1919

3	1	0.13 ∗ C + 0.1419 ∗ BFS + −0.2008 ∗ W + −0.3707 ∗ S + 22.8639
	2	0.116 ∗ C + 0.179 ∗ BFS + 0.0528 ∗ F + −0.155 ∗ W + 0.3605 ∗ S + 20.0435
	3	0.109 ∗ C + 0.0332 ∗ BFS + 0.0323 ∗ F + 0.6567 ∗ S + −0.1772

4	1	0.1002 ∗ C + 0.1175 ∗ BFS + −0.4268 ∗ W + −0.5294 ∗ S + −0.0581 ∗ FA + 124.9081
	2	0.0887 ∗ C + 0.097 ∗ BFS + −0.1639 ∗ W + −0.0381 ∗ CA + −0.0439 ∗ FA + 103.3361
	3	0.1206 ∗ C + 0.0296 ∗ BFS + 1.0002 ∗ S + −2.3015
	4	0.1923 ∗ C + 0.1899 ∗ BFS + 0.1096 ∗ F + 0.385 ∗ S + 0.0802 ∗ CA + 0.0748 ∗ FA + −171.2826

7	1	0.0937 ∗ C + 0.0918 ∗ BFS + 0.0816 ∗ CA + −65.026
	2	0.0843 ∗ C + 0.0893 ∗ BFS + −0.2364 ∗ W + −0.0522 ∗ CA + −0.0542 ∗ FA + 141.3941
	3	0.1069 ∗ C + 0.1079 ∗ W + 1.5966 ∗ S + −16.0794
	4	0.2578 ∗ C + 0.2539 ∗ BFS + 0.176 ∗ F + 0.2456 ∗ W + 0.1289 ∗ CA + 0.1658 ∗ FA + −355.9914
	5	0.1259 ∗ C + −0.3447 ∗ BFS + 0.1478 ∗ F + −0.233 ∗ W + 24.2414
	6	0.2657 ∗ C + 0.2326 ∗ BFS + 0.1769 ∗ F + 0.6028 ∗ S + 0.0627 ∗ CA + 0.123 ∗ FA + −216.9787
	7	0.1446 ∗ C + 0.1444 ∗ BFS + 0.061 ∗ F + 0.6039 ∗ S + 0.1112 ∗ CA + −121.8771

10	1	0.1327 ∗ Cement C + 0.139 ∗ BFS + −0.3442 ∗ Water W + −0.6836 ∗ S + 49.4202
	2	0.1168 ∗ C + 0.046 ∗ BFS + −0.1125 ∗ F + 2.5621 ∗ S + 0.0316 ∗ FA + −36.434
	3	0.1517 ∗ C + 0.1011 ∗ BFS + 0.0631 ∗ F + −16.8066
	4	−0.0346 ∗ BFS + −0.0948 ∗ F + −0.6478 ∗ W + −1.3017 ∗ S + −0.154 ∗ CA + −0.124 ∗ FA + 414.4977
	5	0.0776 ∗ C + 0.0914 ∗ BFS + −0.1966 ∗ W + 44.4496
	6	0.1964 ∗ C + 0.1919 ∗ BFS + 0.1774 ∗ F + 0.9458 ∗ S + 0.087 ∗ CA + 0.0765 ∗ FA + −193.6016
	7	0.0886 ∗ C + 0.0411 ∗ BFS + 0.0647 ∗ F + 0.8154 ∗ S + −1.3541
	8	0.0283 ∗ C + −0.1769 ∗ F + 2.0944 ∗ S + −0.0505 ∗ CA + −0.0596 ∗ FA + 114.1096
	9	0.117 ∗ C + 0.0578 ∗ BFS + 0.1119 ∗ F + −0.4979 ∗ W + 82.2298
	10	−0.1039 ∗ F + −0.4885 ∗ W + −0.0483 ∗ CA + −0.1583 ∗ FA + 297.2198

**Table 10:** Equations for different clusters for estimating concrete compressive strength using LMS regression.

Number of clusters	Cluster	Equation
1	1	0.1123 ∗ C + 0.0829 ∗ BFS + 0.0545 ∗ F − 0.0688 ∗ W + 0.4242 ∗ S + 0.0102 ∗ CA + 0.0043 ∗ FA − 8.3733

2	1	0.1025 ∗ C + 0.0638 ∗ BFS + −0.0386 ∗ F + −0.0068 ∗ W + 0.9019 ∗ S + −0.0088 ∗ CA + −0.0145 ∗ FA + 16.4968
	2	0.1756 ∗ C + 0.1554 ∗ BFS + 0.12 ∗ F + −0.0281 ∗ W + 0.1514 ∗ S + 0.0568 ∗ CA + 0.0588 ∗ FA + −126.1692

3	1	0.1214 ∗ C + 0.1697 ∗ BFS + 0.01 ∗ F + −0.3376 ∗ W + −0.3047 ∗ S + −0.0119 ∗ CA + −0.043 ∗ FA + 93.7417
	2	0.1427 ∗ C + 0.2122 ∗ BFS + 0.0827 ∗ F + −0.1831 ∗ W + 0.3936 ∗ S + 0.0261 ∗ CA + 0.0399 ∗ FA + −42.9607
	3	0.1427 ∗ C + 0.2122 ∗ BFS + 0.0827 ∗ F + −0.1831 ∗ W + 0.3936 ∗ S + 0.0261 ∗ CA + 0.0399 ∗ FA + −42.9607

4	1	0.124 ∗ C + 0.1832 ∗ BFS + 0.005 ∗ F + −0.1905 ∗ W + −0.2358 ∗ S + 0.0784 ∗ CA + 0.0156 ∗ FA + −63.3526
	2	0.0646 ∗ C + 0.0294 ∗ BFS + 0.0119 ∗ F + −0.1295 ∗ W + 0.2192 ∗ S + −0.0479 ∗ CA + −0.0707 ∗ FA + 135.821
	3	0.1357 ∗ C + 0.0365 ∗ BFS + −0.0011 ∗ F + 0.0415 ∗ W + 1.2929 ∗ S + −0.013 ∗ CA + 0.0127 ∗ FA + −13.1736
	4	0.1955 ∗ C + 0.1941 ∗ BFS + 0.1118 ∗ F + 0.0249 ∗ W + 0.4467 ∗ S + 0.0851 ∗ CA + 0.0797 ∗ FA + −186.1104

7	1	0.153 ∗ C + 0.2109 ∗ BFS + −0.0039 ∗ W + −0.0535 ∗ S + 0.0756 ∗ CA + 0.0461 ∗ FA + −131.7132
	2	0.086 ∗ C + 0.0338 ∗ BFS + 0.1355 ∗ F + −0.1872 ∗ W + 0.1889 ∗ S + −0.0453 ∗ CA + −0.057 ∗ FA + 126.286
	3	0.1497 ∗ C + 0.0824 ∗ BFS + −0.0074 ∗ F + 0.254 ∗ W + 1.624 ∗ S + 0.0223 ∗ CA + 0.0439 ∗ FA + −123.2346
	4	0.1313 ∗ C + 0.098 ∗ BFS + −0.0133 ∗ F + −0.119 ∗ W + −0.0542 ∗ S + −0.0312 ∗ CA + −0.0158 ∗ FA + 57.0779
	5	0.1354 ∗ C + −0.1365 ∗ BFS + 0.1906 ∗ F + −0.2073 ∗ W + −0.2973 ∗ S + 0.0069 ∗ CA + 0.0031 ∗ FA + 6.1093
	6	0.2897 ∗ C + 0.1449 ∗ BFS + 0.1695 ∗ F + −0.1257 ∗ W + 0.6174 ∗ S + 0.0505 ∗ CA + 0.103 ∗ FA + −169.8855
	7	0.153 ∗ C + 0.1335 ∗ BFS + 0.0785 ∗ F + 0.0094 ∗ W + 0.6421 ∗ S + 0.1257 ∗ CA + 0.0659 ∗ FA + −198.2299

10	1	0.1655 ∗ C + 0.1993 ∗ BFS + −0.366 ∗ W + −0.6273 ∗ S + 0.0785 ∗ CA + −32.0018
	2	0.0999 ∗ C + 0.0175 ∗ BFS + −0.0505 ∗ F + −0.0565 ∗ W + 1.1747 ∗ S + −0.0327 ∗ CA + −0.0134 ∗ FA + 49.551
	3	0.1137 ∗ C + 0.0975 ∗ BFS + −0.005 ∗ F + −0.0662 ∗ W + 0.3299 ∗ S + −0.0494 ∗ CA + −0.0143 ∗ FA + 64.7487
	4	0.1325 ∗ C + 0.0707 ∗ BFS + −0.0091 ∗ F + −0.1209 ∗ W + −0.0786 ∗ S + −0.0257 ∗ CA + −0.0167 ∗ FA + 55.767
	5	0.0621 ∗ C + 0.082 ∗ BFS + −0.0234 ∗ F + −0.2272 ∗ W + 0.1207 ∗ S + 0.0071 ∗ CA + −0.0091 ∗ FA + 55.3497
	6	0.2211 ∗ C + 0.2119 ∗ BFS + 0.2084 ∗ F + 0.0189 ∗ W + 1.1351 ∗ S + 0.1005 ∗ CA + 0.0905 ∗ FA + −232.357
	7	0.141 ∗ C + 0.102 ∗ BFS + 0.1055 ∗ F + 0.0264 ∗ W + 0.8571 ∗ S + 0.0373 ∗ CA + 0.0708 ∗ FA + −119.3061
	8	0.0156 ∗ C + −0.0279 ∗ BFS + −0.1598 ∗ F + −0.2105 ∗ W + 1.1434 ∗ S + −0.1111 ∗ CA + −0.0991 ∗ FA + 250.545
	9	0.0947 ∗ C + −0.0374 ∗ BFS + 0.0511 ∗ F + −0.0771 ∗ W + 0.615 ∗ S + −0.0546 ∗ CA + −0.0529 ∗ FA + 104.3686
	10	−0.0602 ∗ C + −0.0801 ∗ BFS + 0.083 ∗ F + −0.6562 ∗ W + −2.4912 ∗ S + −0.0621 ∗ CA + 0.0711 ∗ FA + 234.7251

## Appendix

### Equations for Different Clusters for Estimating Compressive Strength Using Various Regression Techniques

See Tables 8, 9, and 10.
